# Compound Odontoma in a 12-Year-Old Boy With Impacted Premolar: A Case Report

**DOI:** 10.7759/cureus.93031

**Published:** 2025-09-23

**Authors:** Eugénie Longé, Anne-Laure Ejeil

**Affiliations:** 1 Department of Oral Surgery, University of Paris Cité, Paris, FRA; 2 Department of Odontology, Bretonneau Hospital, Assistance Publique-Hôpitaux de Paris (AP-HP), Paris, FRA

**Keywords:** case report, cbct, compound odontoma, mandible, odontogenic tumour, paediatric

## Abstract

Odontomas are the most frequent odontogenic tumours and may obstruct the eruption of permanent teeth. A 12-year-old boy was referred for a painless mandibular swelling and absence of tooth 45 on panoramic radiography. Clinical examination showed persistence of 85, cone beam computed tomography (CBCT) revealed a well-circumscribed radiopaque cluster surrounded by a radiolucent rim between 46 and 44, and tooth 45 was displaced lingually. Under local anaesthesia, conservative enucleation was undertaken. Histopathology confirmed a compound odontoma composed of multiple tooth-like structures. Three-month panoramic follow-up showed early bone fill, and at six months, periapical radiography confirmed continued healing. The patient was referred for orthodontic traction/guidance of 45.

## Introduction

Odontomas are benign hamartomatous malformations of odontogenic tissues, usually discovered in the first two decades of life. These benign tumours are mostly formed of enamel and dentin but can also contain a certain amount of pulp and cement tissue. They are classified as compound, composed of multiple miniature tooth-like structures, or complex, consisting of a disorganised mass of dental tissues [1]. They are classically divided into compound lesions (aggregates of multiple miniature tooth-like structures) and complex lesions (disorganised masses of enamel, dentine, cementum and pulp tissue). Compound odontomas predominate in the anterior maxilla, whereas complex odontomas are more frequent in the posterior mandible [2]. Although most lesions are asymptomatic and discovered incidentally, they may cause delayed eruption, tooth displacement or cortical expansion. Early diagnosis and surgical management are essential to avoid functional and aesthetic complications. We report a case of a compound odontoma in a 12-year-old boy, associated with the persistence of the primary mandibular second molar (tooth 85) and failure of eruption of the permanent second premolar (tooth 45). Multidisciplinary management combining surgical and orthodontic approaches was undertaken to facilitate the eruption of tooth 45 and to achieve a functional occlusion for the patient.

## Case presentation

A 12-year-old boy with an unremarkable medical history was referred to the Oral Surgery Department of Bretonneau Hospital for evaluation of a right mandibular swelling and absence of tooth 45, incidentally noted on panoramic radiography. The patient reported no pain or paraesthesia and had not perceived the swelling, suggesting slow growth.

The extraoral examination was unremarkable. Intraoral examination revealed the persistence of the primary mandibular right second molar (85), distal tipping of the mandibular right first premolar (44) and a firm, non-tender buccal bulge extending from 46 to 44, without lingual expansion. There were no signs of infection.

**Figure 1:**
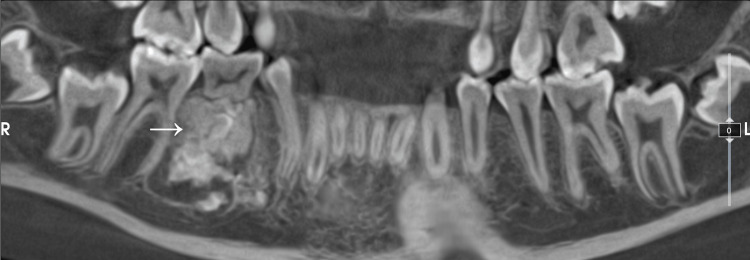
Preoperative CBCT CBCT images showing a radiopaque mass (arrow) surrounded by a radiolucent halo at the apex of tooth 85. It also appears that in this two-dimensional view, tooth 45 was not visible, which explains why the orthodontist was uncertain about its presence. CBCT: cone beam computed tomography

Panoramic radiography demonstrated a well-defined radiopaque mass surrounded by a radiolucent halo, located between teeth 46 and 44. Cone beam computed tomography (CBCT) revealed a lesion measuring 13 × 18 × 11.5 mm, with mild thinning of the buccal cortical plate, without displacement of the inferior alveolar nerve. Distal displacement of tooth 44 and lingual positioning of the developing tooth bud 45 in relation to the lesion were also noted (Figure 1).

The radiographic appearance (clustered radiopacities with radiolucent rim) and clinical context (eruption disturbance and retained 85) supported compound odontoma as the leading diagnosis.

Differential diagnoses considered included ameloblastic fibro-odontoma, cementoblastoma, odontoameloblastoma and other mixed odontogenic lesions.

**Figure 2:**
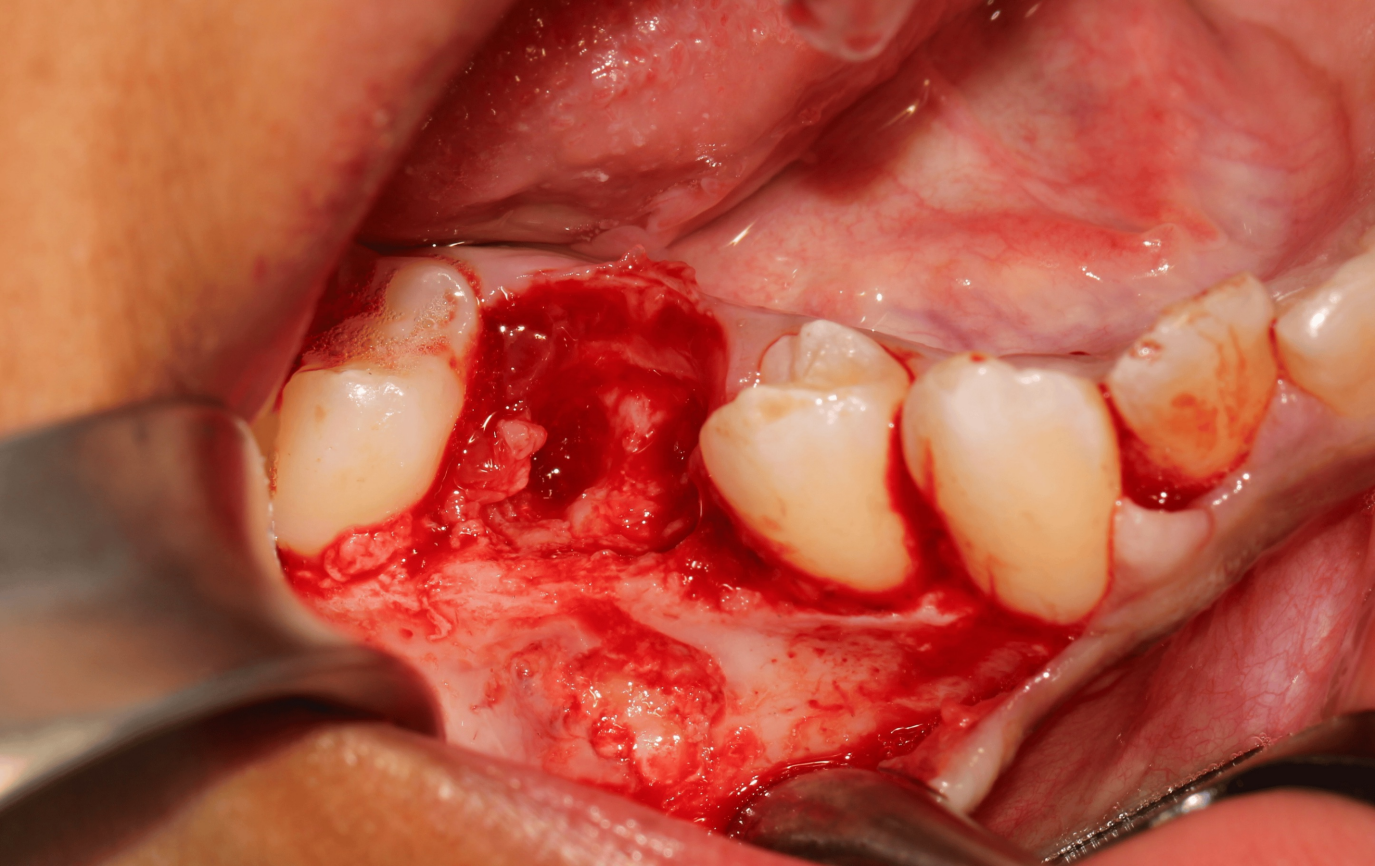
Peroperative views A full-thickness flap was raised from tooth 44 to tooth 46 following the extraction of tooth 85. This view shows a portion of the mass on the buccal cortical plate.

**Figure 3:**
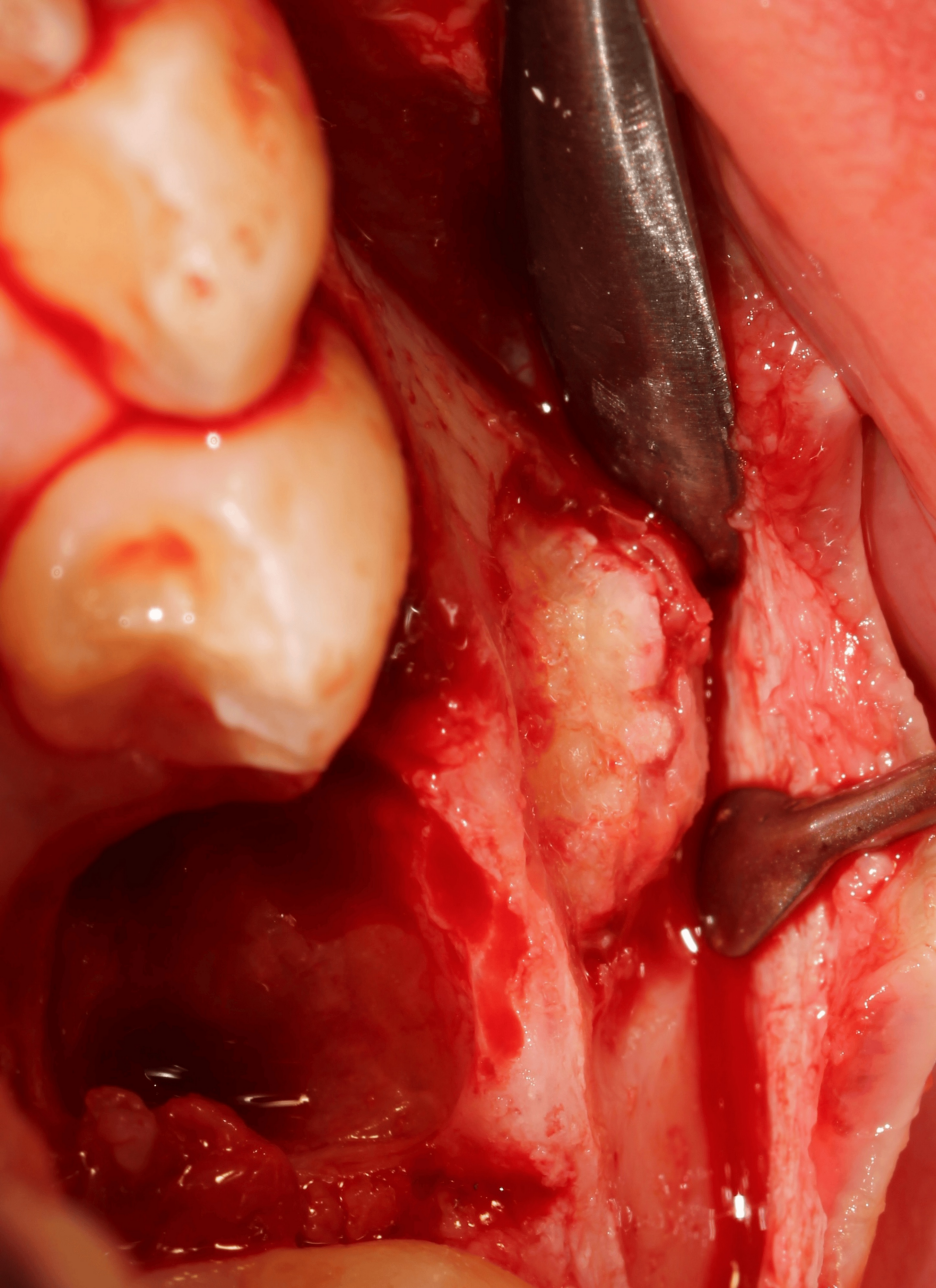
Peroperative views An alveolectomy was performed in the area of the odontoma, visible here in the occlusal view. The lesion appeared to have the same colour as the adjacent teeth.

**Figure 4:**
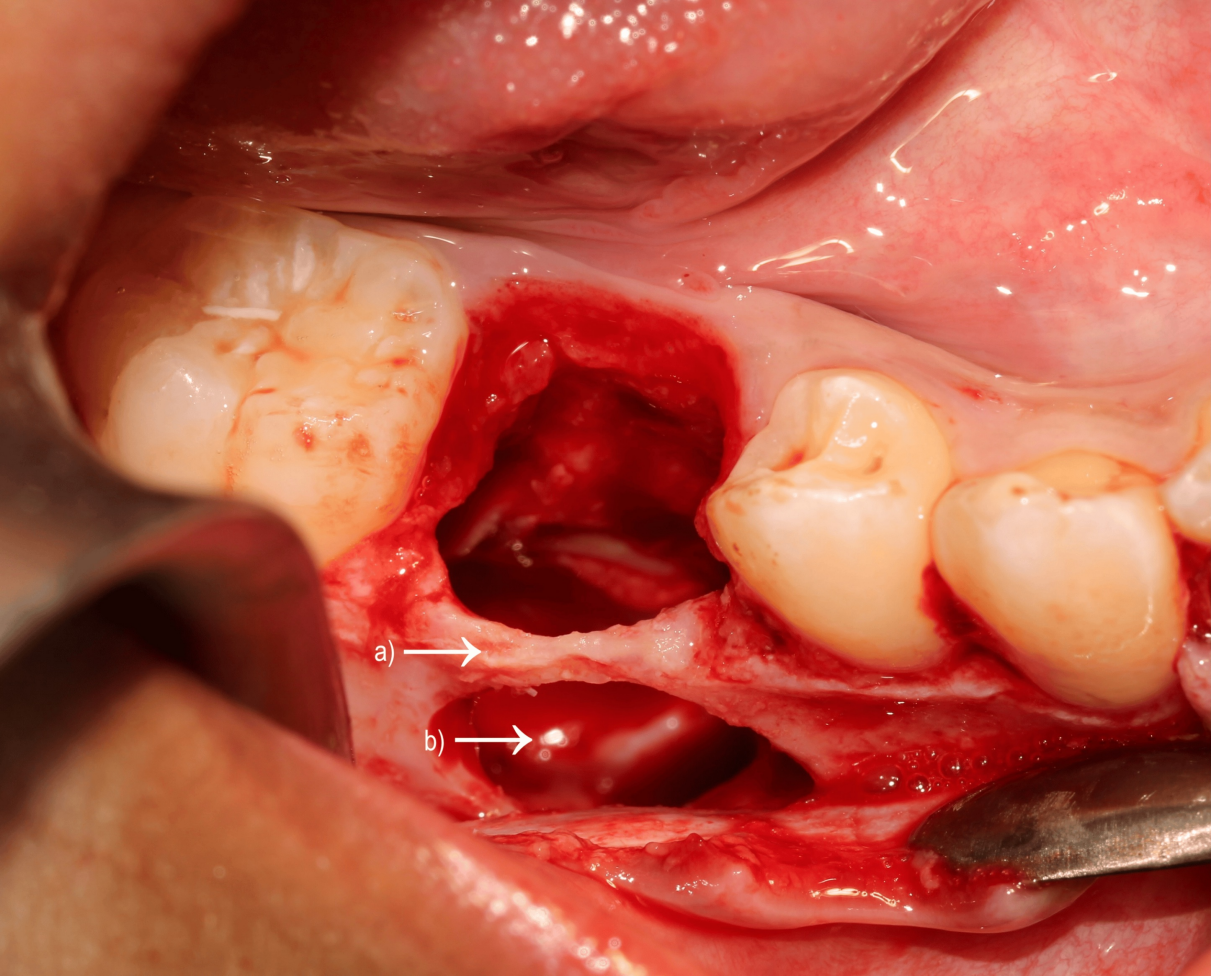
Peroperative views Operative site after the removal of the odontoma showing the empty socket with preservation of the buccal cortical ridge (a) and tooth 45 in apical position (b).

**Figure 5:**
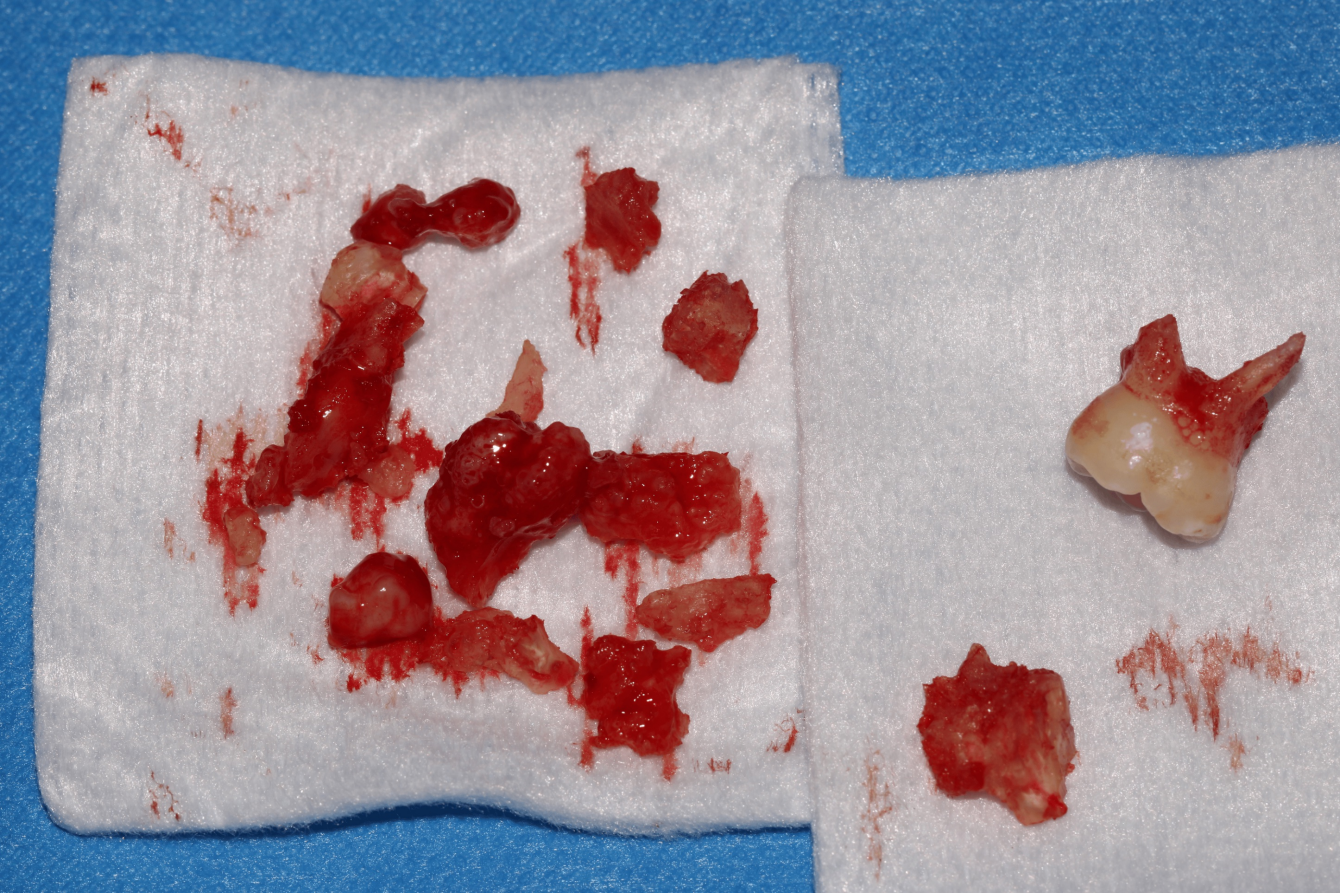
Fragments of the removed odontoma and tooth 85 The odontoma was removed in several fragments because of its size and density, and in order to preserve the buccal cortical ridge.

**Figure 6:**
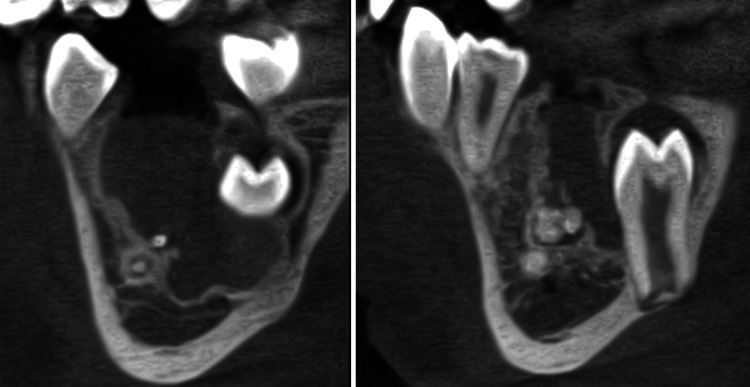
Postoperative CBCT showing the left tissue after the first surgery CBCT performed after the first stage of surgery. In this sagittal view, residual fragments of the odontoma can be observed in the apical region, along with the tooth bud of 45 positioned against the lingual cortical plate. After showing these images to the patient, it was decided to proceed with a second intervention in order to remove as much of the odontoma as possible. CBCT: cone beam computed tomography

Under local anaesthesia (articaine), a conservative enucleation was performed through a buccal approach with careful detachment of the encapsulated mass and preservation of alveolar walls (Figures 2-5). As the apical portion was indistinct from adjacent tissues, only partial removal was safe at the first stage. The odontoma presented as a lesion with a mixed appearance of bone and inflammatory tissue. However, its bone-like colour and density made differentiation from the surrounding bone particularly difficult in the more apical areas, where visibility was reduced. A postoperative CBCT was obtained to map remnants, and after shared decision‑making with the family, a second-stage enucleation removed the residual tissue (Figure 6). The patient was prescribed a one-week course of antibiotics (amoxicillin) following the intervention, in association with an analgesic regimen tailored to his postoperative symptoms. Follow-up appointments were scheduled at one week, three weeks, and three months postoperatively, after which the patient was referred back to his orthodontist.

**Figure 7:**
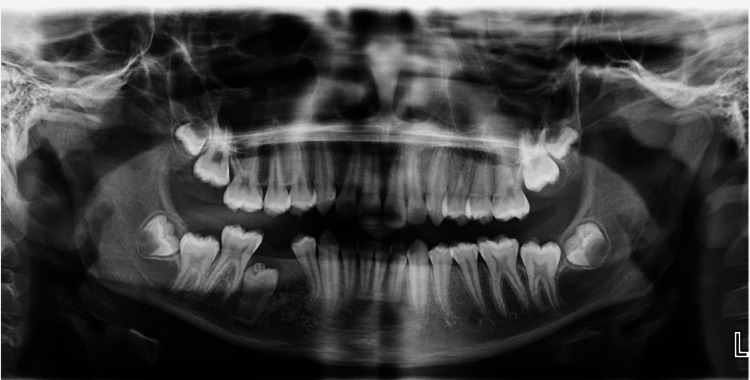
Panoramic radiography three months postoperatively This radiograph is used as a reference image to monitor the eruption of tooth 45.

**Figure 8:**
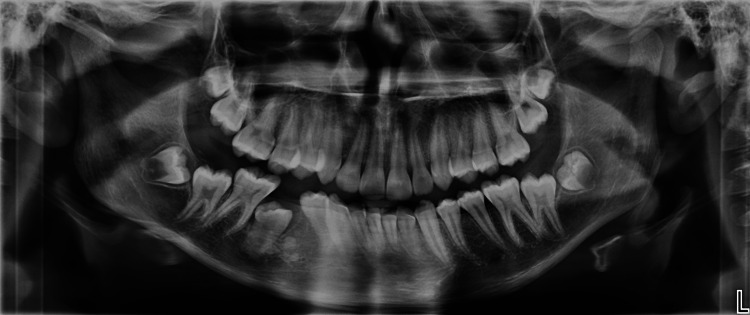
Panoramic radiography six months postoperatively Orthodontic follow-up image

**Figure 9:**
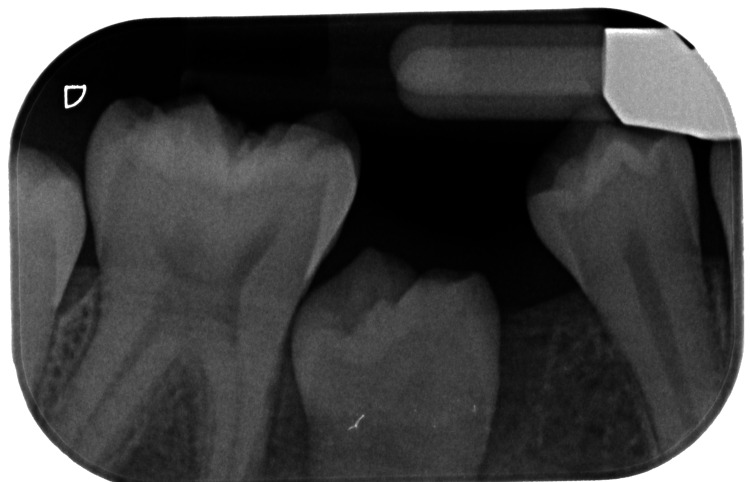
Periapical radiograph at 12 months postoperatively Tooth 45 is slowly erupting.

**Figure 10:**
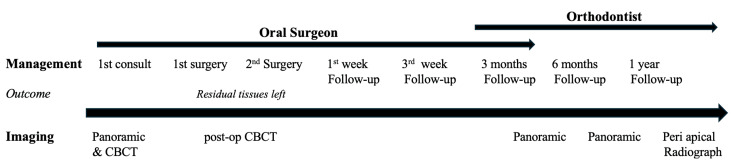
Timeline of case management CBCT: cone beam computed tomography

The follow-up panoramic radiograph was made three months after surgery, showing initial signs of bone healing and providing a reference point for the eruption progress of tooth 45 (Figure 7). The patient was subsequently referred to his orthodontist for continuation of care. A panoramic radiograph (at sixth month) and periapical radiograph (at 12th month) confirmed further bone healing and tooth 45 evolution (Figures 8, 9). Orthodontic treatment will then be initiated to guide eruption and alignment (Figure 10).

## Discussion

Compound odontomas are the most frequent odontogenic tumours, with a reported prevalence of approximately 45.8% [3]. They are currently regarded as hamartomatous malformations. Histologically, they consist of well-differentiated odontogenic tissues arranged in tooth-like structures, often described as denticles. Their most common site is the anterior maxilla, while complex odontomas tend to predominate in posterior mandibular regions. The occurrence of a compound odontoma in the mandibular premolar area, as in the present case, is therefore relatively uncommon [4].

Three forms of odontomas are described in the literature: intraosseous, peripheral and erupted. The present case corresponds to the intraosseous type. All of these variants may occur either as compound or complex odontomas [5].

Compound odontomas are typically diagnosed in the first and second decades of life, often during routine radiographic examinations or in the context of eruption disturbances. For our patient, the lesion was discovered incidentally on a panoramic radiograph at 12 years, with clinical correlation revealing persistence of the primary molar, displacement of the adjacent premolar and buccal cortical expansion. These findings are consistent with the literature. In fact, this one describes eruption delay, tooth displacement and cortical swelling as the most frequent clinical manifestations of odontomas in growing patients [5].

Radiographically, compound odontomas usually display multiple radiopaque masses surrounded by a radiolucent rim, a feature considered pathognomonic. In our case, panoramic imaging revealed a well-defined radiopaque mass with a radiolucent halo [6]. CBCT provided further details on the three-dimensional extent of the lesion, cortical thinning and the relationship with adjacent structures, including the developing second premolar. This highlights the importance of CBCT in treatment planning [7], particularly in paediatric patients, where preserving adjacent tooth buds and the inferior alveolar nerve is a priority. It is an essential tool that has been evaluated as reliable and reproducible in its measurements, allowing for confident surgical planning [8,9].

The aetiology of odontomas remains uncertain, but suggested contributing factors include local trauma, inflammatory or infectious processes of deciduous teeth, genetic mutations and hereditary anomalies [10]. Some studies suggest that this kind of odontogenic tumour could arise from the soft tissue remnants of dental lamina epithelium entrapped within the gingiva [11]. More recently, molecular studies have implicated dysregulation of odontogenic signalling pathways, such as WNT/β-catenin, in their pathogenesis [12].

Differential diagnoses include ameloblastic fibro-odontoma, cementoblastoma, odontoameloblastoma [13], fibroma [14] and peripheral giant cell granuloma.

The treatment of choice is conservative surgical enucleation [1], which is curative in most cases. In the present case, complete removal required two surgical sessions, as the initial intervention could not fully separate the lesion from the surrounding apical tissues. Postoperative follow-up showed progressive bone healing on panoramic [15] and periapical radiographs documenting recovery. Importantly, the patient was referred for orthodontic management to guide the eruption and alignment of the permanent dentition, illustrating the interdisciplinary approach often required in young patients.

Prognosis after complete excision is excellent, with recurrence being extremely rare. However, timely intervention remains critical, as delayed treatment may increase the risk of malposition [6], prolonged orthodontic therapy or even resorption of adjacent roots [16]. Our case demonstrates that even in situations requiring staged surgery, conservative management can achieve favourable outcomes, preserving both function and aesthetics.

## Conclusions

Compound odontomas are benign odontogenic malformations that can interfere with the eruption of permanent teeth. This case illustrates the importance of early diagnosis, careful surgical management and interdisciplinary follow-up to ensure optimal functional and aesthetic outcomes in young patients. CBCT proved valuable in assessing the lesion’s extent and its relationship with adjacent anatomical structures, thereby guiding surgical planning. The postoperative evaluation conducted between the two surgical procedures would not have allowed for a clear identification of the residual position of the odontoma without the use of CBCT imaging. The presence of apical remnants could have compromised the proper eruption and alignment of tooth 45 within the dental arch, potentially interfering with the overall orthodontic treatment plan. The isolated loss of tooth 85, without appropriate surgical management of the odontoma, could have resulted in exacerbated distal migration of tooth 44, potentially interfering with proper arch development and future occlusal alignment. Timely intervention not only prevents complications such as tooth displacement or prolonged orthodontic treatment but also contributes to preserving mandibular growth and occlusal harmony.
